# Synthesis of a Conjugated D-A Polymer with Bi(disilanobithiophene) as a New Donor Component

**DOI:** 10.3390/molecules21060789

**Published:** 2016-06-17

**Authors:** Makoto Nakashima, Yousuke Ooyama, Takuya Sugiyama, Hiroyoshi Naito, Joji Ohshita

**Affiliations:** 1 Department of Applied Chemistry, Graduate School of Engineering, Hiroshima University, 1-4-1 Kagamiyama, Higashi-Hiroshima 739-8527, Japan; makoshima59@gmail.com (M.N.); yooyama@hiroshima-u.ac.jp (Y.O.); 2 Department of Physics and Electronics, Graduate School of Engineering, Osaka Prefecture University, 1-1 Gakuen-cho, Naka-ku, Sakai, Osaka 599-8531, Japan; takuya.sugiyama.oe@pe.osakafu-u.ac.jp

**Keywords:** bulk heterojunction, polymer solar cell, D-A polymer, disilanobithiophene

## Abstract

A new conjugated donor-acceptor (D-A) polymer **pDSBT2-BT** containing bi(disilano-bisthiophene) and benzothiadiazole as donor and acceptor units, respectively, was prepared. The polymer showed a broad UV-vis absorption band at λ_max_ = 599 nm in chlorobenzene. The absorption band was shifted to λ_max_ = 629 nm when the polymer was measured as a film, indicating enhanced interchain interactions of the polymer. Bulk hetero-junction polymer solar cells (BHJ-PSCs) were fabricated using **pDSBT2-BT** and PC_71_BM as host and guest materials, respectively. Optimization of cell fabrication conditions provided a maximal power conversion efficiency of 3.3% and the following cell parameters: *V*_oc_ = 0.86 V, *J*_sc_ = 7.56 mA/cm^2^, and FF = 0.51. Although the efficiency still leaves much to be desired, these data underscore the potential of **pDSBT2-BT** as a high-voltage polymer solar cell material.

## 1. Introduction

**Figure 1:**
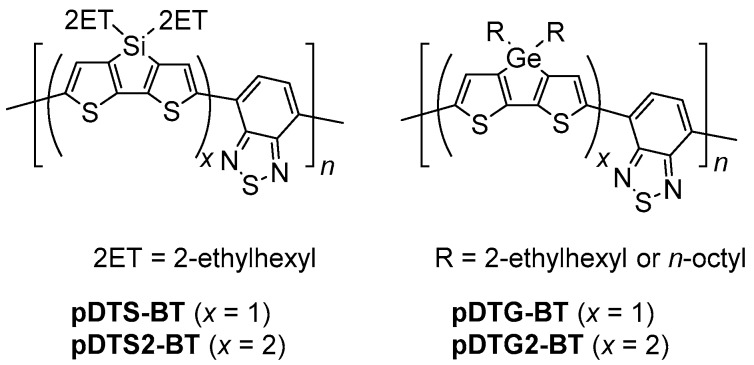
Dithienosilole- and dithienogermole-containing D-A polymers.

Bulk heterojunction polymer solar cells (BHJ-PSCs) are of current interest because of their potential applications in lightweight and flexible modules and simple fabrication by a solution process. In this system, a blend film of an electron-donating host polymer and an electron-accepting guest material, such as PC_61_BM and PC_71_BM, is employed as the active layer. As the host polymer, conjugated donor-acceptor (D-A) polymers have been extensively studied [1,2]. D-A polymers usually show red-shifted broad absorptions arising from rather strong intramolecular D-A electronic interactions, making it possible to utilize the wide wavelength range of sunlight. Intermolecular D-A interactions are also expected in the solid state, which enhances the carrier-transport properties of the polymers. Dithienosiloles [3,4,5,6,7] and dithienogermoles [8,9,10] have been studied as the donor components of D-A polymers. These highly planar tricyclic systems show extended conjugation. The flexibility of the substituents on the Si and Ge atoms with C-Si and C-Ge bonds that are longer than C-C and C-N bonds provides sufficient solubility, without suppressing the intermolecular interactions significantly. Recently, it has been also demonstrated that D-A polymers with bi(dithienosilole) [5] and bi(dithienogermole) [11] as donor units (**pDTS2-BT** and **pDTG2-BT**, Figure 1) show higher carrier-transport properties than the corresponding dithienosilole- and dithienogermole-containing analogs (**pDTS-BT** [3] and **pDTG-BT** [9], Figure 1), rendering them applicable as active materials for organic transistors and PSCs with enhanced performance.

Recently, we reported the synthesis of conjugated D-A polymers with disilanobithiophene (DSBT) as the new donor unit and demonstrated the potential applications of these polymers to high-voltage polymer solar cells [12,13]. In these polymers, twisting of the bithiophene unit in DSBT and between the DSBT donor and acceptor units lowers the HOMO energy levels of the polymers. In this work, we prepared a bis(disilanobithiophene)-benzothiadiazole (**pDTBT2-BT**) alternating polymer and examined its potential as a host material for BHJ-PSCs. The open-circuit voltage of the cell was higher than that based on a similar alternating polymer **pDSBT-BT** [12,13], reported previously. 

## 2. Results

### 2.1. Synthesis

**Scheme 1:**
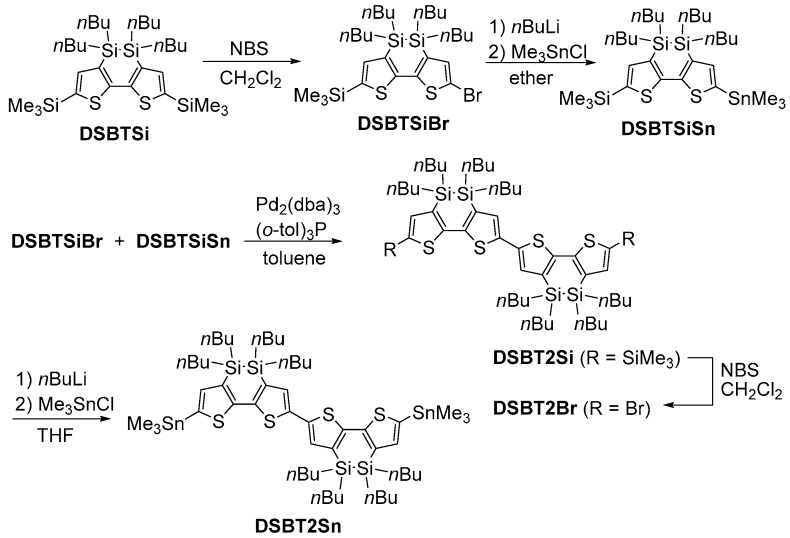
Synthesis of bis(disilanobithiophene) monomer.

For the preparation of **pDSBT2-BT**, distannylbi(disilanobithiophene) (**DSBT2Sn**) was employed as the monomer (Scheme 1). Monobromination of disilyldisilanobithiophene **DSBTSi** with one equiv of NBS proceeded selectively to provide bromo(trimethylsilyl)disilanobithiophene **DSBTBrSi**. Stannylation of **DSBTBrSi** gave **DSBTSiSn**, and subsequent Stille coupling of **DSBTSiBr** with **DSBTSiSn** in toluene under reflux yielded bis(trimethylsilyl)disilanobithiophene **DSBT2Si**. **DSBT2Si** underwent bromination and then stannylation to afford **DSBT2Sn** in 24% yield in five steps from **DSBTSi**.

**Scheme 2:**
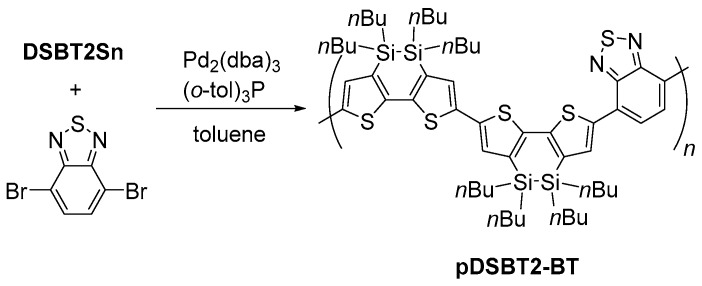
Polymer synthesis.

The Stille coupling of DSBT2Sn with dibromobenzothiadiazole in toluene at 70 °C for 5 days, followed by reprecipitation of the resulting organic products gave **pDSBT2-BT** as a dark purple solid in 53% yield (Scheme 2). The polymer **pDSBT2-BT** was soluble in toluene, chloroform, and chlorobenzene, and slightly soluble in THF and hexane, but insoluble in ethyl acetate, methanol, and ethanol. The polymer molecular weight was determined by gel permeation chromatography (GPC) to be *M*_n_ = 13,000 (*M*_w_/*M*_n_ = 1.9), relative to polystyrene standards. The polymer structure was confirmed from its ^1^H- and ^13^C-NMR spectra. As can be seen in Figure S2, the ^1^H-NMR spectrum has three singlets due to the aromatic CH protons, together with those of butyl groups on silicon atoms. The proton integration ratio was in good agreement with the regular structure shown in Scheme 2.

### 2.2. Optical and Electrochemical Properties of **pDSBT2-BT**

**Figure 2:**
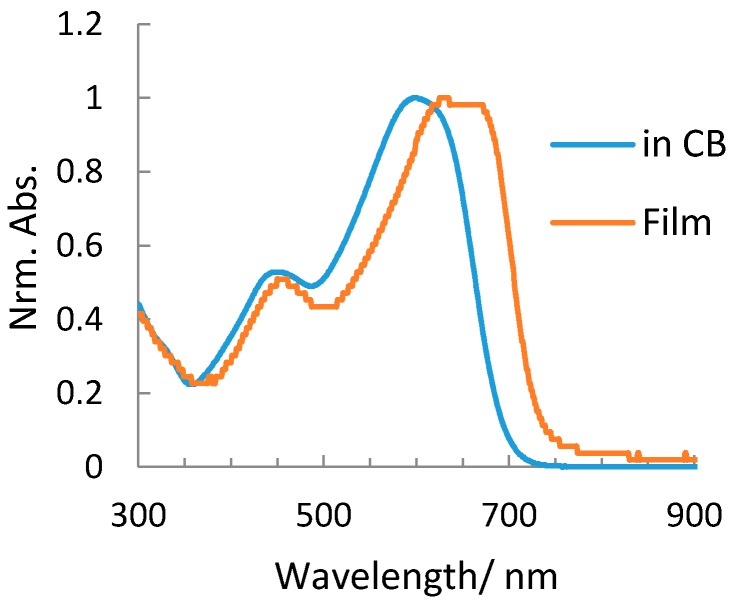
UV absorption spectra of **pDSBT2-BT** in chlorobenzene and in film form.

**Figure 3:**
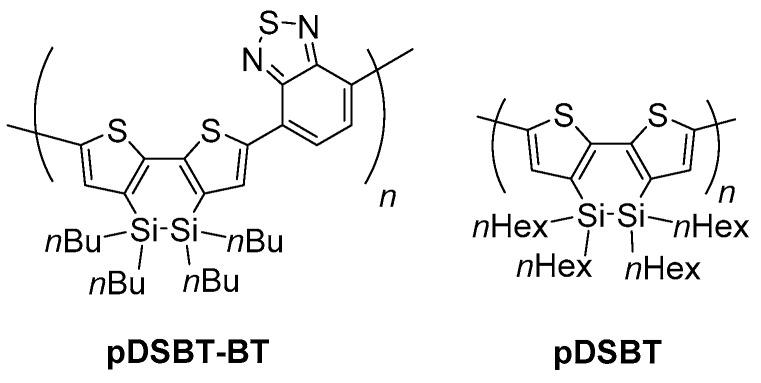
Structures of DSBT- and DTG-containing polymers.

Figure 2 shows the UV absorption spectra of **pDSBT2-BT** in solution and film, and Table 1 summarizes the data of **pDSBT2-BT** and those of **pDSBT-BT** [12] and homopolymer **pDSBT** [14] reported previously (Figure 3).

The absorption was shifted to the longer wavelength region when the spectrum was measured as a film, suggesting enhanced interchain interaction and/or planarity of the polymer in film. Compared with the data of **pDSBT-BT** and **pDSBT**, **pDSBT2-BT** has a HOMO-LUMO energy gap in between those of **pDSBT** and **pDSBT-BT** in both solution and film. The degree of tendency to form aggregates could be also estimated from the UV-vis spectra. For **pDSBT-BT**, a shoulder peak ascribed to the aggregated polymer chains was observed even in the solution spectrum, and this peak was enhanced in the film spectrum. The present **pDSBT2-BT** showed a peak for the polymer aggregation only in the film spectrum. **pDSBT**, in contrast, showed no polymer aggregation peaks even in the film spectrum, but the peak in the film spectrum was broadened and slightly red-shifted compared with that in solution. These results indicate that the formation of aggregates is enhanced in the order of **pDSBT** < **pDSBT2-BT** < **pDSBT-BT**.

A polymer film containing tetrabutylammonium perchlorate (TBAP) was cast on a platinum working electrode and its cyclic voltammogram (CV) was measured in acetonitrile with TBAP as the electrolyte. The HOMO energy level of **pDSBT2-BT**, which was estimated on the basis of the CV oxidation onset (*E*_HOMO_/eV = −4.8 − [*E*_onset_ − *E*_1/2_(Fc/Fc^+^)]/V), was the same as that of **pDSBT-BT**, as listed in Table 1 [12,13], thus indicating that the larger HOMO-LUMO gap of **pDSBT2-BT** than that of **pDSBT-BT** was primarily ascribed to the higher-lying LUMO. This is likely due to the low content of acceptor BT units in **pDSBT2-BT**. 

**Figure 4:**
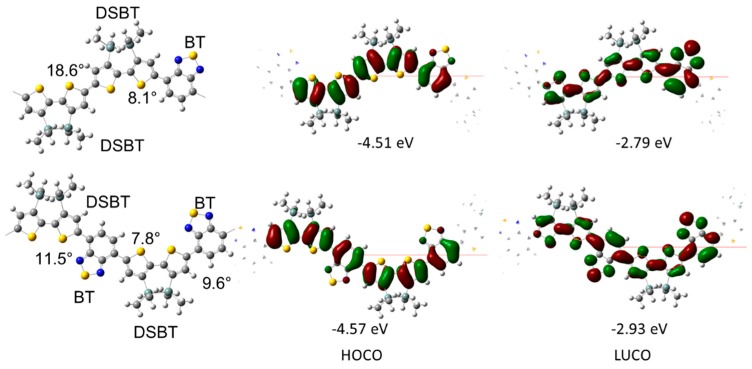
Optimized geometries of the unit cells with interunit twisting angles and HOCO and LUCO profiles and their energy levels for **pDSBT2-B**T (**top**) and **pDSBT-BT** (**bottom**).

To understand the electronic state of **pDSBT2-BT**, we carried out crystal orbital (CO) calculations on the polymer model that was simplified by replacing the butyl groups on the silicon atoms by methyl groups at the level of B3LYP/6-31G(d,p) on the Gaussian 09 program. The optimized geometry of the unit cell and the profiles and the energy levels of the highest occupied crystal orbital (HOCO) and the lowest unoccupied crystal orbital (LUCO) are presented in Figure 4. The geometry possesses an almost planar structure, with the small twisting angles between the DSBT and BT units, leading to the efficient conjugation in the polymer backbone. This is in contrast to that previously reported for **pBSBT-BT** that was computed using a DSBT-BT unit cell to have a lager twisting angle of 84.8° between DSBT and BT units [13]. We therefore recalculated the **pDSBT-BT** model with the use of a lager unit cell of DSBT-BT-DSBT-BT and obtained a more planar structure similar to that of the **pDSBT2-BT** model (Figure 4). This is likely due to rather flat potential surface of the polymers with respect to the twisting angles and the planarity readily changes depending on the computation conditions. For both the **pDSBT2-BT** and **pDSTB-BT** models, the HOCOs are delocalized over the bithiophene and phenylene units, but the thiadiazole rings exert little contribution. On the other hand, the LUCOs are rather localized on the BT units, suggesting possible charge separation at the photo excited states. Disilane σ- and σ*-orbitals are not obviously included in the HOCOs and LUCOs. The model of **pDSBT-BT** possesses a smaller HOCO-LUCO energy gap, in accordance with the experimental observations described above.

### 2.3. Device Fabrication

BHJ-PSCs were fabricated with the structure of ITO/PEDOT:PSS/**pDSBT2-BT**:PC_71_BM/ETL/Al (ETL = electron transport layer) and the active area of 0.04 cm^2^. First, we examined the dependence of cell performance on the ratio of **pDSBT2-BT**:PC_71_BM in the active layer. 

**Figure 5:**
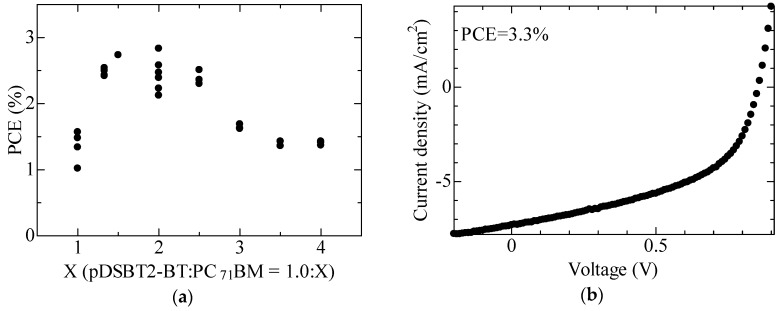
Plots of PCE of **pDSBT2-BT**-based PSCs *vs.* **pDSBT2-BT**:PC_71_BM ratio (**a**); and *J*-*V* plots of PSC that yielded the best performance in the present study (**b**).

As shown in Figure 5a (ETL = Ca), the 1:2 ratio gave the best performance. Next, the effects of ETL were investigated in cells with **pDSBT2-BT**:PC_71_BM = 1:2. Ca and LiF with different layer thicknesses were examined as ETL (Table 2, runs 1–5). 

The best performance was obtained for the cell with LiF (0.5 nm) (run 3), although use of Ca (5 nm) as ETL gave similar results (run 1) with a slightly higher *V*_oc_ and a lower *J*_sc_. Attempts to improve cell performance by annealing the active layer (runs 6 and 7) and using 1,8-diiodooctane as the processing additive (runs 8 and 9) were unsuccessful. Finally, the thickness of the active layer was optimized (runs 10–12) to give a maximal PCE of 3.3% (run 10, Figure 5b). The introduction of an inverted structure of ITO/ZnO/PEI/**pDSBT2-BT**: PC_71_BM/MnO_3_/Al (PEI = polyethyleneimine) resulted in a decrease of both current density and voltage of the cell (run 13). When the performance of the present PSCs with **pDSBT2-BT** was compared with that of **pDSBT-BT**-based PSCs reported previously (*V*_oc_ = 0.74 V, *J*_sc_ = 7.30 mA/cm^2^ for ETL = Ca [12]; *V*_oc_ = 0.82 V, *J*_sc_ = 12.69 mA/cm^2^ for ETL = LiF [13]), the present PSCs with **pDSBT2-BT** showed a higher *V*_oc_ and a lower *J*_sc_. The lower *J*_sc_ was likely due to the weaker polymer interchain interaction of **pDSBT2-BT** than that of **pDSBT-BT**, although the reason for the higher *V*_oc_ was not clearly understood. This contrasted the fact that **pDTG2-BT**-based PSCs showed higher *J*_sc_ and lower *V*_oc_ than those of **pDTG-BT**, as reported in the literature [11].

## 3. Materials and Methods

### 3.1. General

The polymerization was carried out under a dry argon atmosphere. Toluene used as reaction solvent was distilled from calcium hydride and stored over activated molecular sieves before use. NMR spectra were measured on Varian 400-MR and Varian System 500 spectrometers (Agilent Technologies, Santa Clara, CA, USA). UV-vis absorption spectra were measured on a SHIMADZU-UV-3150 spectrometer (Shimadzu, Kyoto, Japan). GPC was carried out using serially connected Shodex KF2001 and KF2002 columns (Showa Denko, Tokyo, Japan) and THF as the eluent. 

### 3.2. Device Fabrication

For the fabrication of BHJ-PSCs, patterned ITO glasses were washed sequentially with acetone and 2-propanol under ultrasonication and the glasses were further cleaned by exposure to UV/ozone. A PEDOT-PSS layer (Clevios P VP AI 4083, Heraeus Precious Metals, Leverkusen, Germany) was then formed by spin-coating at 3000 rpm on the ITO glasses followed by baking at 130 °C for 10 min in air. The substrates were transferred to a glove box under a dried nitrogen atmosphere (dew point: −80 °C), and a chlorobenzene solution of **pDSBT2-BT**:PC_71_BM blend was spin-coated on the PEDOT-PSS layer at 1500 rpm for 60 s. After drying *in vacuo*, Ca or LiF, and Al (50 nm) were vapor-deposited through a shadow mask (active area was 0.04 cm^2^) on the organic active layer at the base pressure of 6 × 10^−4^ Pa. BHJ-PSCs were encapsulated under nitrogen. The vapor deposition and the encapsulation were successively carried out in the glove box. BHJ-PSCs were tested in air with a computer-programmed Keithley 2611 source meter (TFF, Tokyo, Japan) under a solar simulator (Asahi Spectra HAL-320, Asahi Spectra, Tokyo, Japan) that simulated AM1.5 solar irradiance with the power density of 100 mW/cm^2^.

### 3.3. Preparation of **pDSBT2-BT**

A mixture of DSBT_2_Sn (0.242 g, 0.198 mmol), 4,7-dibromo-2,1,3-benzothiadiazole (5.83 × 10^−2^ g, 0.198 mmol), Pd_2_(dba)_3_ (9.10 × 10^−3^ g, 9.90 × 10^−3^ mmol), (*o*-tolyl)_3_P (1.21 × 10^−2^ g, 3.96 × 10^−2^ mmol), and toluene (18 mL) was stirred at 70 °C for 5 days. The resulting mixture was allowed to cool to room temperature and an aqueous solution (30 mL) of sodium *N*,*N*-diethyldithiocarbamate trihydrate (3.1 g) was added. The mixture was heated to 80 °C for 2 h. The organic layer was separated and washed with water, 3 vol % acetic acid aqueous solution, and then water again, in that order. The organic layer was dried over anhydrous magnesium sulfate and the solvent was removed under vacuum. The residue was reprecipitated using a sequence of solvent mixtures: toluene/methanol, toluene/ethanol, and toluene/ethyl acetate, to provide 108 mg (53% yield) of **pDSBT2-BT** as a dark purple solid: m.p. >300 °C; ^1^H-NMR (δ in C_6_D_4_Cl_2_, 500 MHz) 0.87–0.93 (m, 24H), 0.96–1.20 (m, 16H), 1.34–1.46 (m, 16H), 1.50–1.62 (m, 16H), 7.48 (s, 2H), 7.74 (s, 2H), 8.39 (s, 2H); ^13^C-NMR (δ in CDCl_3_, 125 MHz) 12.87, 13.49, 13.57, 26.55, 26.62, 27.26, 27.32, 125.28, 134.38, 135.39, 135.63, 136.34, 137.86, 144.78, 146.96, 152.54; GPC *M*_n_ 13,000, *M*_w_ 24,700, *M*_w_/*M*_n_ 1.9; UV-vis abs λ_max_ 599 nm (in C_6_H_5_Cl).

## 4. Conclusions

We prepared **pDSBT2-BT** as the first D-A polymer with bi(disilanobithiophene) as the donor. The polymer showed photovoltaic applications in BHJ-PSCs. Although the cell PCE was not very high, the relatively high *V*_oc_ of the cell seemed to indicate the high potential of the polymer. Studies to improve *J*_sc_ by optimizing the polymer structure through changing of the substituents of the silicon atoms and the acceptor structure are in progress, and the results will be reported elsewhere.

## Figures and Tables

**Table 1:** Polymer properties ^a^.

Polymer	*M*_n_ (*M*_w_/*M*_n_) ^b^/gmol^−1^	UV-vis abs λ_max_/nm		*E*_g_^c^/eV	*E*_HOMO_^d^/eV	*E*_LUMO_^e^/eV
		Solution	Film			
**pDSBT** ^f^	7200 (2.0)	542	nd ^g^	1.9	nd ^g^	
**pDSBT-BT** ^h^	20,000 (1.9)	633, 680	650, 689	1.7	−5.2	−3.5
**pDSBT2-BT**	13,000 (1.9)	599	629, 668	1.8	−5.2	−3.4

^a^ After reprecipitation; ^b^ Determined by GPC relative to polystyrene standards; ^c^ Optical HOMO-LUMO gap; ^d^ Estimated from CV onset; ^e^ *E*_g_ + *E*_HOMO_; ^f^ Reference [14]; ^g^ Not determined; ^h^ Reference [12].

**Table 2:** Performance of **pDSBT2-BT**-based BHJ-PSCs.

Run	Cell Type ^a^	ETL (Thickness/nm)	Annealing Temp/°C	Additive (/vol %)	Active Layer Thickness/nm	*V*_oc_/V	*J*_sc_ /mAcm^−2^	FF	PCE/%
1	Con	Ca (5)	non	non	70	0.86	6.91	0.48	2.8
2	Con	Ca (25)	non	non	70	0.81	4.04	0.35	1.2
3	Con	LiF (0.5)	non	non	70	0.85	7.03	0.49	2.9
4	Con	LiF (1.0)	non	non	70	0.85	5.63	0.40	1.9
5	Con	LiF (1.5)	non	non	70	0.85	6.29	0.45	2.4
6	Con	LiF (0.5)	50	non	70	0.85	5.57	0.43	2.0
7	Con	LiF (0.5)	80	non	70	0.84	5.47	0.42	1.9
8	Con	LiF (1.5)	non	DIO ^b^ (1)	70	0.52	5.14	0.40	1.1
9	Con	LiF (1.5)	non	DIO ^b^ (2.5)	70	0.33	2.06	0.26	0.2
10	Con	LiF (0.5)	non	non	60	0.86	7.56	0.51	3.3
11	Con	LiF (0.5)	non	non	50	0.87	7.14	0.48	3.0
12	Con	LiF (0.5)	non	non	40	0.88	6.33	0.46	2.6
13	Inv					0.79	5.99	0.42	2.0

^a^ Con: ITO/PEDOT:PSS/**pDSBT2-BT**:PC_71_BM (1:2)/ETL/Al (ETL = electron transport layer); Inv: ITO/ZnO/PEI/**pDSBT2-BT**:PC_71_BM (1:2)/MnO_3_/Al (PEI = polyethyleneimine); ^b^ 1,8-Diiodooctane, used as a processing additive in **pDSBT2-BT**:PC_71_BM solutions in chlorobenzene/THF.

## Supplementary Materials

Supplementary materials can be accessed at: http://www.mdpi.com/1420-3049/21/6/789/s1.
