# Supplementary material for: Synthesis of a Conjugated D-A Polymer with Bi(disilanobithiophene) as a New Donor Component
Source: Molecules. 2016 Jun 17;21(6):789. doi: 10.3390/molecules21060789 (PMC6273805; doi:10.3390/molecules21060789)
Supplement: Supplementary file 1 [file molecules-21-00789-s001.pdf]

Supplementary Materials: Synthesis of a Conjugated D-A Polymer with Bi(disilanobithiophene) as a New Donor Component

Makoto Nakashima, Yousuke Ooyama, Takuya Sugiyama, Hiroyoshi Naito and Joji Ohshita

1. Experimental

1.1. General

All reactions were carried out under a dry argon atmosphere. THF, ether, dichloromethane, and toluene that were used as the reaction solvents were distilled from calcium hydride and were stored over activated molecular sieves before use. Monomer 4,4,5,5-tetra-*n*-butyl-2,7-bis(trimethylsilyl)-dithieno[3,2-*c*:2′,3′-*e*]disila-cyclo-hexadiene (**DSBTSi**) [1] was prepared according to literature procedure. Other materials were commercially available and used without further purification. NMR spectra were measured on Varian 400-MR and Varian System 500 spectrometers (Agilent Technologies, Santa Clara, CA, USA). APCI mass spectra were measured on a Thermo Fisher Scientific LTQ Orbitrap XL spectrometer (Thermo Fisher Scientific, Waltham, MA, USA) at N-BARD Hiroshima University, and EI mass spectra were recorded on a Shimadzu QP-2020A spectrometer (Shimadzu, Kyoto, Japan).

1.2. Preparation of ***DSBTSiBr***

To a solution of 2.75 g (4.64 mmol) of **DSBTSi** in 50 mL of dichloromethane was added 0.785 g (4.41 mmol) of *N*-bromosuccinimide (NBS) in a few portions at 0 °C and the resulting mixture was stirred for 1 h. The mixture was hydrolyzed with water and the organic layer was washed with sodium thiosulfate (aq), water, and brine. The organic layer was dried over anhydrous potassium carbonate. After evaporation of the solvents, the residue was purified by chromatography on silica gel with hexane to give 2.52 g (4.25 mmol, 91% yield) of **DSBTBr** as yellow viscous oil: EI-MS *m*/*z* 598 [M$^+$]; ^{1}H-NMR (δ in $CDCl_3$, 400 MHz) 0.33 (s, 9H), 0.77–0.95 (m, 20H), 1.27–1.34 (m, 16H), 6.95 (s, 1H), 7.11 (s, 1H); ^{13}C-NMR (δ in C_6D_6, 100 MHz) −0.03, 12.83, 13.03, 13.85, 13.88, 26.94, 26.95, 27.43, 27.60, 111.01, 134.34, 135.04, 136.01, 136.11, 139.61, 140.50, 150.57; HR-MS (APCI) Calcd for $C_{27}H_{47}BrS_2Si_3$: [M$^+$] 598.16049. Found: 598.16162.

1.3. Preparation of ***DSBTSiSn***

To a solution of 1.90 g (3.17mmol) of **DSBTSiBr** in 30 mL of ether was added drop wise 2.06 mL (3.20 mmol) of a 1.55 M *n*BuLi solution in hexane at −80 °C. After stirring the mixture for 30 min, 0.695 g (3.48 mmol) of trimethyltin chloride was added. The resulting mixture was allowed to warm to room temperature and stirred overnight. After hydrolysis with ice-water, the organic layer was separated and the aqueous layer was extracted with hexane. The organic layer and the extracts were combined and washed with water and brine. The organic layer was dried over anhydrous potassium carbonete. After evaporation of the solvents, the residue was purified by preparative GPC eluting with toluene to give 1.60 g (2.31 mmol, 73% yield) of **DSBTSiSn** as dark yellow viscous oil: EI-MS *m*/*z* 684 [M$^+$]; ^{1}H-NMR (δ in $CDCl_3$, 400 MHz) 0.32 (s, 9H), 0.38 (s, 9H),0.78–0.94 (m, 20H), 1.26–1.35 (m, 16H), 7.09 (s, 1H), 7.13 (s, 1H); ^{13}C-NMR (δ in C_6D_6, 125 MHz) −8.41, 0.07, 13.24, 13.30, 13.90, 13.91, 26.98, 27.00, 27.02, 27.71, 27.73, 133.81, 134.02, 136.77, 138.96, 140.58, 141.57, 152.25, 152.52; HR-MS (APCI) Calcd for $C_{30}H_{56}S_2Si_3Sn$: [M$^+$] 684.21477. Found: 684.21576.

1.4. Preparation of ***DSBT2Si***

A mixture of 2.62 g (4.37 mmol) of **DSBTSiBr**, 2.99 g (4.37 mmol) of **DSBTSiSn**, 0.200 g (0.218 mmol) of $Pd_2(dba)_3$, 0.266 g (0.874 mmol) of (*o*-tolyl)$_3$P, and 45 mL of toluene was vacuum degassed and heated at 70 °C for 6 h. The resulting mixture was allowed to cool down to room temperature. The mixture was poured into ice-water, and the organic layer was separated and the aqueous layer was

extracted with hexane. The organic layer and the extracts were combined and washed with water and brine. The organic layer was dried over anhydrous potassium carbonate. After evaporation of the solvents, the residue was purified by chromatography on alumina with hexane to give 2.77 g (2.67 mmol, 61% yield) of **DSBT2Si** as yellow solid: m.p. 158.7–159.7 °C; HR-MS (APCI) *m*/*z* 1038 [M^+]; ^{1}H-NMR (δ in $CDCl_3$, 400 MHz) 0.34 (s, 18H), 0.81–0.94 (m, 40H), 1.30–1.35 (m, 32H), 7.08 (s, 2H), 7.14 (s, 2H); ^{13}C-NMR (δ in $CDCl_3$, 100 MHz) 0.22, 12.63, 12.70, 13.84, 26.74, 26.80, 27.17, 27.21, 130.09, 134.37, 134.88, 135.02, 138.25, 140.58, 144.55, 150.60; HR-MS (APCI) Calcd for $C_{54}H_{94}S_4Si_6$: [M^+] 1038.48485. Found: 1038.48608.

1.5. Preparation of ***DSBT2Br***

To a solution of 2.77 g (2.67 mmol) of **DSBT2Si** in 30 mL of dichloromethane was added 0.950 g (5.33 mmol) of *N*-bromosuccinimide (NBS) in a few portions at 0 °C and the resulting mixture was stirred for 2 h. The mixture was hydrolyzed with water and the organic layer was washed with sodium thiosulfate (aq), water, and brine. The organic layer was dried over anhydrous potassium carbonete. After evaporation of the solvents, the residue was purified by chromatography on silica gel with hexane to give 2.60 g (2.47 mmol, 92% yield) of **DSBT2Br** as orange solid: m.p. 134.8–135.9 °C; HR-MS (APCI) *m*/*z* 1050 [M^+]; ^{1}H-NMR (δ in C_6D_6, 400 MHz) 0.81–0.99 (m, 40H), 1.27–1.46 (m, 32H), 7.04 (s, 2H), 7.34 (s, 2H); ^{13}C-NMR (δ in $CDCl_3$, 125 MHz) 12.53, 12.59, 13.80, 13.82, 26.74, 27.11, 27.17, 110.01, 130.21, 135.04, 135.10, 135.14, 135.91, 143.39, 146.95; Anal Calcd for $C_{48}H_{76}Br_2S_4Si_4$: C, 54.72; H, 7.27. Found: C, 54.61; H, 7.34.

1.6. Preparation of ***DSBT2Sn***

To a solution of 1.69 g (1.60mmol) of **DSBT2Br** in 32 mL of THF was added drop wise 1.21 mL (3.22 mmol) of a 2.65 M *n*BuLi solution in hexane at −80 °C. After stirring the mixture for 30 min, 0.670 g (3.37 mmol) of trimethyltin chloride was added. The resulting mixture was allowed to warm to room temperature and stirred overnight. After hydrolysis with ice-water, the organic layer was separated and the aqueous layer was extracted with hexane. The organic layer and the extracts were combined and washed with water and brine. The organic layer was dried over anhydrous potassium carbonete. After evaporation of the solvents, the residue was purified by preparative GPC eluting with toluene to give 1.27 g (1.04 mmol, 65% yield) of **DSBT2Sn** as yellow solid: HR-MS (APCI) *m*/*z* 1222 [M^+]; ^{1}H-NMR (δ in $CDCl_3$, 500 MHz) 0.39 (s, 18H), 0.81–0.93 (m, 40H), 1.29–1.36 (m, 32H), 7.07 (s, 2H), 7.10 (s, 2H); ^{13}C-NMR (δ in $CDCl_3$, 125 MHz) −7.90, 12.65, 12.79, 13.84, 26.77, 26.80, 27.19, 27.24, 130.01, 133.98, 134.31, 134.92, 135.83, 141.62, 144.70; HR-MS (APCI) Calcd for $C_{54}H_{94}S_4Si_4Sn_2$: [M^+] 1222.33539. Found: 1222.33720.

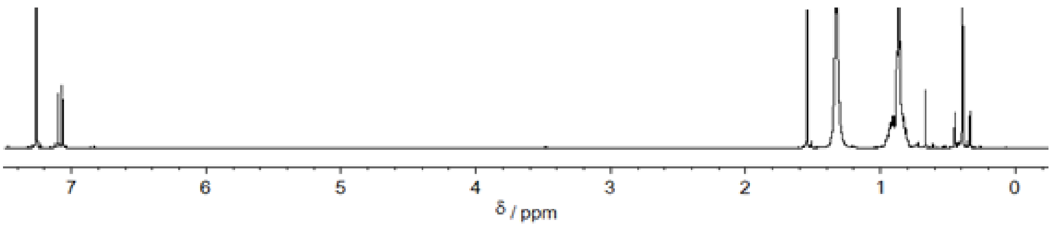

Figure S1. ^{1}H-NMR spectra of **DSBT2Sn**.

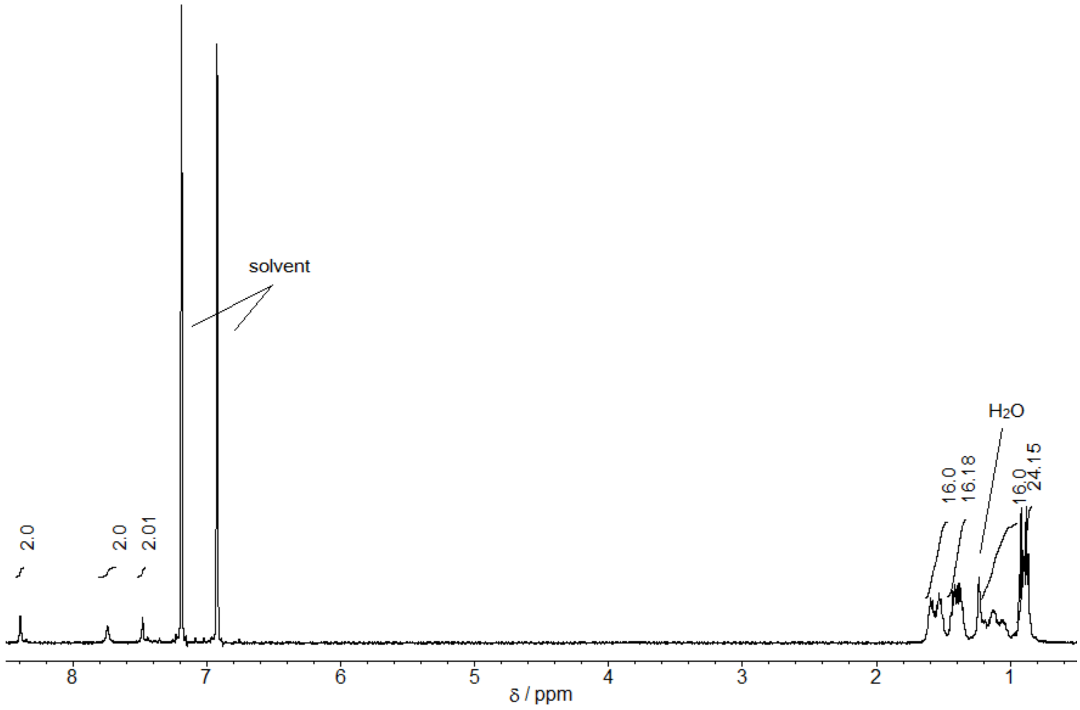

Figure S2. ^{1}H-NMR spectrum of **pDSBT2-BT**.

Reference

1. Ohshita, J.; Nakashima, M.; Tanaka, D.; Morihara, Y.; Fueno, H.; Tanaka, K. Preparation of a D-A polymer with disilanobithiophene as a new donor component and application to high-voltage bulk heterojunction polymer solar cells. *Polym. Chem.* **2014**, *5*, 346–349.
